# Can arterial wave augmentation in young adults help account for variability of cardiovascular risk in different British ethnic groups?

**DOI:** 10.1097/HJH.0000000000001066

**Published:** 2016-08-19

**Authors:** Luca Faconti, Maria J. Silva, Oarabile R. Molaodi, Zinat E. Enayat, Aidan Cassidy, Alexis Karamanos, Elisa Nanino, Ursula M. Read, Philippa Dall, Ben Stansfield, Seeromanie Harding, Kennedy J. Cruickshank

**Affiliations:** a Diabetes & Nutritional Sciences Division, King's College London; Cardiovascular and Social Epidemiology Groups, London; b MRC/CSO Social and Public Health Sciences Unit, University of Glasgow; c Institute for Applied Health Research, Glasgow Caledonian University, Glasgow, UK

**Keywords:** augmentation index, cardiovascular risk, ethnicity, vascular stiffness

## Abstract

Traditional cardiovascular risk factors do not fully account for ethnic differences in cardiovascular disease. We tested if arterial function indices, particularly augmentation index (AIx), and their determinants from childhood could underlie such ethnic variability among young British adults in the ‘DASH’ longitudinal study.

**Methods:**

DASH, at http://dash.sphsu.mrc.ac.uk/, includes representative samples of six main British ethnic groups. Pulse wave velocity (PWV) and AIx were recorded using the Arteriograph device at ages 21–23 years in a subsample (*n* = 666); psychosocial, anthropometric, and blood pressure (BP) measures were collected then and in two previous surveys at ages 11–13 years and 14–16 years. For *n* = 334, physical activity was measured over 5 days (ActivPal).

**Results:**

Unadjusted values and regression models for PWVs were similar or lower in ethnic minority than in White UK young adults, whereas AIx was higher – Caribbean (14.9, 95% confidence interval 12.3–17.0%), West African (15.3, 12.9–17.7%), Indian (15.1, 13.0–17.2%), and Pakistani/Bangladeshi (15.7, 13.7–17.7%), compared with White UK (11.9, 10.2–13.6%). In multivariate models, adjusted for sex, central SBP, height, and heart rate, Indian and Pakistani/Bangladeshi young adults had higher AIx (β = 3.35, 4.20, respectively, *P* < 0.01) than White UK with a similar trend for West Africans and Caribbeans but not statistically significant. Unlike PWV, physical activity, psychosocial or deprivation measures were not associated with AIx, with borderline associations from brachial BP but no other childhood variables.

**Conclusion:**

Early adult AIx, but not arterial stiffness, may be a useful tool for testing components of excess cardiovascular risk in some ethnic minority groups.

## INTRODUCTION

Britain, like many Western countries, has a significant and rapidly growing ethnically diverse population, well prior to arrivals of refugees from Middle Eastern wars. Recent census data [1] show that the White European ethnic group accounted for 86.0% of the usual resident population in England and Wales in 2011, decreasing from 91.3% in 2001 and 94.1% in 1991. Meanwhile, over the last two decades, ethnic minority groups (South Asian, African-Caribbean, and Black African) continued to rise, and in metropolitan areas like London, proportions increased up to 40% of the total.

These population data may continue to have long-term influences on health profiles, particularly ethnic differences in cardiometabolic disease, noted over the last 40 years [2].

Cardiovascular disease (CVD) is the leading cause of death worldwide [3] with increasing rates globally, in part related to ageing. Mortality from coronary heart disease (CHD) and stroke in South Asian migrants to the United Kingdom is between 50 and 100% higher than the White British population [4]; conversely, people of black African and African Caribbean origin are still significantly protected from CHD, although mortality from stroke is even higher than in South Asians [4]. Higher cardiovascular risk in migrant populations is not just a British issue [5,6]; similar results were obtained recently in Norway where South Asian migrants had increased risks of myocardial infarction and stroke compared with the resident population, and stroke was more common in people from sub-Saharan Africa and Southeast Asia [7]. Very similar data were also published for migrants to the Netherlands [8,9]. These remarkable ethnic differences cannot be fully explained by traditional cardiovascular and metabolic risk factors, such as hypertension, dyslipidemia, central adiposity, or insulin resistance, measured in midlife [10].

As arterial stiffness has become a major intermediary outcome for cardiovascular events and mortality [11–13], it could be a useful tool to investigate ethnic patterns of CVD. Further, arterial function indices could be early targets for intervention at a stage of life when traditional cardiovascular risk factors only weakly predict later disease and mortality. Not only pulse wave velocity (PWV) but arterial wave reflections [14,15] have emerged as important markers of vascular health and predict cardiovascular risk independent of conventional risk factors, including blood pressure (BP); however, data regarding arterial indices in ethnic minorities are rare.

Here we examine how far central augmentation index (AIx), the most widely used index of wave reflections, may underlie ethnic differences in cardiovascular risk among a multiethnic British population cohort – the ‘DASH’ longitudinal study.

## METHODS

Details of the DASH study can be found at http://dash.sphsu.mrc.ac.uk/ and in a published cohort profile [16]. The sample was recruited between 2002 and 2003, from 51 secondary schools in 10 London boroughs. A total of 6643 students, aged 11–13 years, took part in the baseline survey. In 2005–2006, 4782 (88% of children in 49 schools, 72% of the cohort, aged 14–16 years, took part in the first follow-up. A 10% subsample of (*N* = 666, 97% participation rate) took part in a pilot follow-up study, which was completed in March 2014. Response rates (≥90% of the invited pilot sample) were similar by ethnicity and sex. The subsample consisted of 107 White UK, 102 Black Caribbeans, 132 Black Africans, 99 Indian, 111 Pakistani/Bangladeshi, and 115 other (mainly mixed) ethnicities, and chosen to be representative by sex and socioeconomic status across the 10 London boroughs and 51 schools.

The study was approved by the NHS Research Ethics Committees. Written informed consent was obtained from participants. Ethnicity in DASH was measured by self-reported ethnicity, checked against reported parental ethnicity and grandparents’ country of birth. Bangladeshis and Pakistanis were combined because of relatively small sample sizes.

### Physical measures

Measurement protocols can be found at http://dash.sphsu.mrc.ac.uk and were taken from the World Health Organization manual.

SBP and DBP were measured using validated OMRON M5-I semiautomatic devices and appropriately sized cuffs, after the participant had sat quietly for a timed 5 min, with more than 1 min between three subsequent readings. The mean of the second and third readings was used in analysis, as previously reported [17–19] At 21–23 years, PWV, central AIx, central SBP (cSBP), and brachial BP were also measured using the Arteriograph 24-h device, previously calibrated and standardized [20]. The device records up to eight cardiac cycles, three separate times in one sitting. The aortic path length is measured with a long arm caliper, from suprasternal notch to pubic rami.

Physical activity was not measured in detail during adolescence. In the follow-up, a subsample of participants *N* = 334, 76% of those invited, wore a waterproofed ActivPal monitor continuously for 5 days. Worn on the front of the thigh, the monitor is valid for identifying sitting standing and walking [21]. The following were derived and reported per day: steps taken, upright time, time walking at more than 100 steps/min (equivalent to moderate–vigorous physical activity), sit-to-stand transitions, and proportion of daytime sitting (between 0900 and 2100 h) spent in prolonged (>20 min) bouts.

### Social measures

A self-administered questionnaire measured other social factors, including health behaviors, racism, and socioeconomic circumstances (SECs). Reported racism was assessed using standardized questions on ‘unfair treatment ’on the grounds of race, skin color, country of birth, or religion in various locations (school, street, work, etc.) [22].

In adolescence, SEC was measured through parental employment plus the family affluence scale based on number of cars, computers, holidays, etc. [23]. In adulthood, SEC was measured through own education and employment.

### Statistical method

The core model for AIx contained heart rate, height, cSBP (at 21–23y), sex, and ethnicity. We first examined the influence of current exposures at 21–23 years, and then tested the influence of similarly measured exposures at ages 11–13 years, by adding them to the core model. Each variable was tested in univariate models (added to the core model) before final multivariable linear regressions were conducted; all modeling were performed using Stata 13 (Stat Corp. LP, College Station, Texas, USA). Statistical significance was considered at *P* value <0.05.

## RESULTS

As we previously reported [24], unadjusted PWVs in Black Caribbean and White UK young men were similar (mean + SD 7.9 + 0.3 vs. 7.6 + 0.4 m/s) and lower in other groups at similar SBPs (120 mmHg) and BMIs (24.6 kg/m^2^). Furthermore, in fully adjusted models all ethnic minorities had lower (or similar) values of PWV compared with White UK young people. Two physical activity measures (number of steps/day and time walking >10 000 steps/day), were also negatively associated with PWV [24].

**FIGURE 1:**
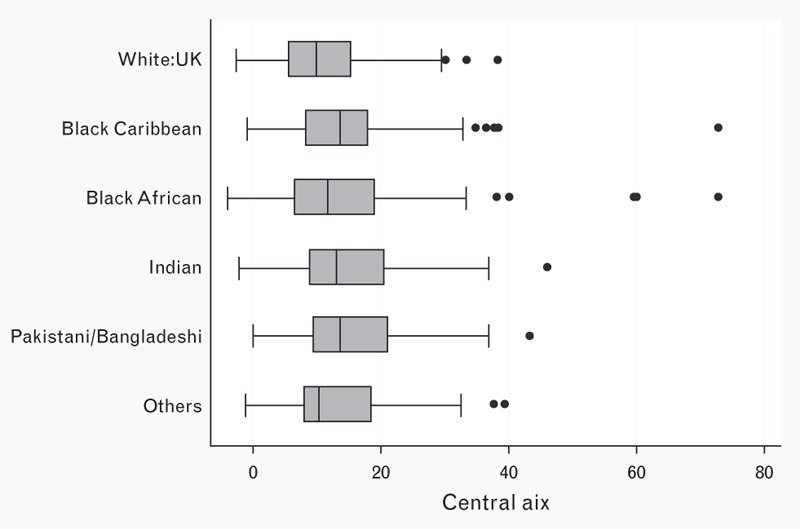
Augmentation index and ethnicity at 21–23 years. The determinants of adolescent, now young adults, social well-being and health study. Central AIx, augmentation index.

Focusing on wave reflection, unadjusted AIx values were higher in women (mean, 95% confidence interval 16.2, 15–17.3%) compared with men (12.5, 11.3–13.6%; *P* < 0.001) and in all ethnic minority groups – Indian (15.1, 13.0–17.2%), Pakistani/Bangladeshi (15.7, 13.7–17.7%), Black Caribbean (14.9, 12.3–17.0%), and Black African (15.3, 12.9–17.7%) – compared with White UK (11.9, 10.2–13.6%), even if borderline significant (*P* = 0.07; Fig. 1). Univariate analysis showed significant correlations between the index of wave reflection and ethnic minorities (all *P* < 0.05); however, factors which are known to have an influence on AIx showed major differences too: South Asians (i.e. both Indian and Pakistani/Bangladeshi groups) were shorter and had lower SBP compared with White UK and other minorities, whereas BA and Black Caribbean tended to have lower heart rates compared with others (Table 1).

Therefore, all these variables were included in the core model where sex, heart rate, height, and cSBP were strongly associated with AIx (all *P* < 0.001). Focusing on ethnicity, Indian and Pakistani/Bangladeshi continued to have significantly higher AIx values compared with other groups after fully adjusting for confounding factors (respective β = 3.35 and 4.20, both *P* < 0.005; Table 2). AIx was also borderline significantly higher in Black African (β = 2.65, *P* = 0.047), including adjustments for childhood variables, and only in Black Caribbean it remained marginally but not significantly higher than in White UK peers.

No influence was detectable for body composition parameters or ‘fat’ such as BMI (*P* = 0.5), waist-to-height ratio (*P* = 0.4) or overweight status (*P* = 0.2) so these were not included in the core model. Regarding BP, DBP (*P* = 0.001), mean arterial pressure (MAP; *P* = 0.025), cSBP (*P* < 0.001) but not brachial SBP (*P* = 0.679) were positively related to AIx; using MAP instead of cSBP in the core model (not shown) did not change any correlations. Psychosocial or deprivation measures also were not correlated with AIx (and their inclusion did not change any previous associations); further adjustments of the core model for 11–13 years variables only found a borderline (negative) association with adolescent MAP (*P* = 0.055).

Of the subsample (*n* = 415) with objective ActivPal measures, both men and women spent some 70% of waking hours sedentary so overall only about 36 min/day in moderate–vigorous activity. No variable associated with physical activity emerged as related to AIx after inclusion in the core model (not shown).

## DISCUSSION

The present study showed clear ethnic differences in AIx, considered an index of wave reflection, in a multiethnic cohort living in similar areas, addressing an issue on which little is published.

Previous small studies in adults showed conflicting results regarding ‘White’ and ‘Black’ young adults in the United States and Europe, respectively [25,26]. Another found that differences in AIx between 94 East Asian and 47 age-matched White peers disappeared after adjusting for height [27]. In large population-based studies throughout the world (10 550 adults), after adjustment for age, heart rate, MAP, and body size, black Africans had markedly higher AIx than British Whites [28]. In 2057 adults aged 21–90 years with type 2 diabetes in the multiethnic state of Singapore, AIx was significantly higher in Indians (28.1 ± 10.8%) than Chinese (26.1 ± 10.7%, *P* < 0.002) [29]. Epidemiological data in the same area found that CHD and mortality was higher in the Indian population compared with the Chinese one [30,31] supporting the idea that arterial stiffness and wave reflection may, at least partially, underlie the differences in such adverse outcome.

In our multi ethnic cohort living in the London area, PWV was similar or lower in ethnic minorities compared with White UK [24], whereas AIx was significantly higher in Indian and Bangladeshi/Pakistani than White UK even after adjustment for confounding factors (sex, heart rate, height, and cSBP, – Table 2). These results may suggest that the parameter of wave reflection, particularly in young South Asian individuals, could indicate important ethnic differences in central hemodynamics, which cannot be fully assessed with conventional sphygmomanometry.

Previously, the general view has been that brachial BP outcomes were ‘more severe’, or ‘worse’ in black populations, even at given levels of BP. However, there remains some doubt whether this is and was so in unbiased genuinely representative population samples (now difficult to find) or after full adjustment for increased hypertension prevalence and other, including social, factors. That view, long considered here [32,33], was substantiated in the long-term follow-up of US studies [34], and recently in the long-term UK ‘SABRE’ study [35]. In the latter three-dimensional echo study, cardiac remodeling rather than hypertrophy was found to be the main issue.

It seems possible that wave reflections, here as AIx, may contribute to the development of left ventricular hypertrophy or ’remodeling’ and could explain the higher rates of hypertensive target organ damage in some ethnic minorities, as was found in African-Americans and in African-Caribbeans in Britain [36–38]. Left ventricular hypertrophy in nonathletic people reflects subclinical organ damage and is very likely an early indicator of cardiovascular, especially hypertensive heart disease.

Taking these considerations together, it seems that in ethnic minorities, and particularly in South Asians, increased central wave reflection from early adulthood could be a determinant of target organ damage before the onset of other well established risk factors.

In such context, discrepancy between the results of PWV and AIx is not surprising as AIx is not a surrogate marker of PWV [39]. Rather, as a measure of arterial wave reflection, it probably depends both on the speed of the pressure wave and on anatomical and mechanical characteristics of the arterial tree. These latter characteristics may be influenced by the vascular tone of the small muscular arteries and arterioles rather than by the elastic properties of the aorta [40].

In our analysis, SBP was not correlated with AIx, likely in part explained by characteristic aortic pressure amplification in young people. In the elderly, the reflected waves return to the aorta during systole, thereby increasing SBP and pulse pressure; in contrast, in younger people, reflected pulse waves return during diastole, resulting in an increase in mean DBP [41].

Thus, in our final model we used only cSBP and MAP. Having outlined these concepts, some traditional some not, we should say that the origins of the ‘augmentation’ are by no means certain. Both amplified Windkessel-like effects and excess (aortic) ‘reservoir’ pressure may be additional, or even replacement, causes of these apparent wave reflections [42,43]. Recently, in patients undergoing cardiac catheterization the reduction in augmentation pressure after nitroglycerin administration was, at least in part, dependent on ventricular contraction/relaxation dynamics rather than reflection effects [44].

In the core model, no ‘fat parameters’ were added because of lack of significant correlation in univariate analysis. Although arterial stiffness and vascular remodeling are generally greater in obese compared with nonobese individuals [45], this may be because of co-occurrence of cardiovascular risk factors rather than obesity *per se*. DeVallance *et al.*[46] found in a small population (102 adults) without type 2 diabetes and CVD that AIx increased in men but decreased in women with increasing BMI; contrarily, in a diabetic cohort, no correlation was found between BMI and augmentation pressure [47].

Psychosocial or deprivation measures did not show any correlation with AIx (and their inclusion in the model did not change any previous associations); further adjustments of the core model for 11–13 years variables only underlined a borderline (negative) association with adolescent MAP (*P* = 0.055).

As previously described in DASH [24], SBP became higher in men than women at 14–16 years and there was a marked increase in this sex difference at 21–23 years with mean SBP hardly changing in women. However, at the age of 11–13 years, SBP was similar between Black African, Black Caribbean, and White UK and lower for Pakistani/Bangladeshi and Indian (*P* < 0.005), with no ethnic differences for DBP, suggesting that growth acceleration maybe a critical period in which a ‘delayed maturation’ of the cardiovascular system may, potentially, be a risk factor for development of CVD. Clearly, the influence of childhood BP on AIx in later life needs further investigation, considering that in our populations no data on cSBP and AIx were collected at the age of 11–13 years. Finally, we found no association between AIx and parameters of physical activity in contrast with our previous data on PWV [24].

Beneficial effects of physical activity on arterial stiffness are found, and across various populations [48–50], probably related to arterial stiffening resulting from both passive (increased intima–media thickness) and active (smooth vascular muscle tone) components, both of which can be altered by physical activity [51]. However, our results for AIx are in line with the Framingham analysis (2376 participants, mean age 47 years) [52] which did not show any effect of physical activity, measured continuously for 8 days, on AIx nor on flow-mediated dilatation. Those data suggest that physical activity could be associated with attenuated age-related increases in intima–media thickness, with very small effects on the active components of vascular muscle tone and endothelial dysfunction. These suggestions again need further investigation in younger people, free from CVD.

In conclusion, a multiethnic cohort of young adults living in the same area both PWV and AIx showed ethnic differences; however, only AIx was higher in ethnic minorities compared with White UK. These data suggest that parameters of wave reflection, rather than arterial stiffness, may underlie ethnic differences at this age and could be a useful tool for testing components of excess cardiovascular risk.

### Limitations

Limitations include a relatively small sample size (about 100 per ethnic group, balanced by sex, and smaller numbers with physical activity measured) and its cross-sectional nature so far.

Another is how far AIx is a measure of reflection as there may be components of arterial compliance and reservoir function as well as or rather than wave reflection *per se*[53]. A study comparing the relationship between carotid AIx and wave reflection indices from wave intensity and wave separation analysis across adult ages in 65 healthy people suggested AIx gave misleading result, especially for its negative values [54]. However, this analysis was a relatively small sample (*n* = 65) done on carotid arteries.

Relatively little data on prognostic value of PWV and AIx measured with Arteriograph device are available. Only a few follow-up studies evaluated prediction by the Arteriograph's stiffness and wave reflection parameters on cardiovascular events or mortality with conflicting results. Akkus *et al.*[55] reported that arterial stiffness and wave reflection measured by it can predict further cardiovascular events in patients after myocardial infarction and the same group [56] showed that AIx and PWV predicted mortality independent of other variables in a small population of advanced heart failure patients. A follow-up study in CKD found no prognostic value for Arteriograph PWV and AIx on cardiovascular mortality in hemodialysis [57].

The prognostic value of PWV and AIx measured with oscillometric devices in a wider range of clinical conditions and in young populations free from CVD are needed.

## ACKNOWLEDGEMENTS

We acknowledge the invaluable support of participants and their parents, the Participant Advisory Group, schools, civic leaders, local GP surgeries and community pharmacies, the Clinical Research Centre at Queen Mary University of London, the Clinical Research Facility at University College Hospital, the survey assistants and nurses involved with data collection, the Primary Care Research Network, and Professors Sanders and Cruickshank at the Diabetes and Nutritional Sciences Division at Kings College London for hosting the feasibility study. S.H. is the principal investigator of DASH. All authors contributed to study design, analyses, and writing of the article. The study was funded by the Medical Research Council (MC_U130015185/MC_UU_12017/1/MC_UU_12017–13.), Chief Scientist Office (SPHSU13) and North Central London Research Consortium and the Primary Care Research Network.

The study was funded by the MRC (MC_U130015185/MC_UU_12017/1-13), Chief Scientist Office (SPHSU13), North Central London Research Consortium and the Primary Care Research Network.

### Conflicts of interest

There are no conflicts of interest.

## Figures and Tables

**TABLE 1:** Descriptive profile of the sample (95% confidence interval) by ethnic groups. The determinants of adolescent, now young adults, social well-being and health study

	Ethnicity					
Variable	White UK	Black Caribbean	Black African	Indian	Pakistani/Bangladeshi	Others
AIx (%)	11.9 (10.2–13.6)	15.3 (12.9–17.7)^*^	14.7 (12.4–17.0)^*^	15.1 (13.0–17.2)^*^	15.7 (13.7–17.7)^*^	13.2 (11.5–14.9)
Heart rate (bpm)	70.1 (68.1–72.2)	66.8 (65.0–68.6)^*^	67.2 (65.2–69.2)^*^	70.1 (68.0–72.3)	71.3 (69.3–70.2)	68.4 (66.7–70.2)
Height (cm)	172.3 (170.7–174.1)	167.1 (165.3–168.9)	166.5 (163.5–169.5)^**^	161.9 (160.0–164)^**^	163.3 (161.6–165)^**^	166.1 (163.7–168.4)^**^
BMI (kg/m^2^)	23.8 (23–24.7)	26.1 (25–27.2)^**^	25.9 (25–26.8)^**^	23.8 (22.8–24.9)	24.3 (23.5–25.2)	24.5 (23.7–25.3)
SBP (mmHg)	120.5 (117.7–123.2)	119.7 (117.2–122.3)	118.7 (116.5–120.9)	115.9 (113.5–118.3)^**^	115 (113–117.2)^**^	115.2 (113.2–117.1)^**^
DBP (mmHg)	71 (69.2–72.8)	70.9 (68.9–72.7)	70.1 (68.5–71.7)	67.8 (66.1–69.5)^**^	67.5 (66.1–68.9)^**^	68.1 (66.6–68.9)^*^
cSBP (mmHg)	108.9 (106.4–111.4)	109.1 (106–112.1)	107.6 (105.2–110)	104.8 (102.2–107.3)^*^	105.2 (102.9–107.5)^*^	104.4 (102.3–106.7)^**^
MAP (mmHg)	87.5 (85.4–89.5)	87.1 (85.1–89.1)	86.3 (84.6–88)	83.8 (82–85.6)^**^	83.3 (82–85.6)^**^	83.7 (82.2–85.2)^**^

AIx, augmentation index; cSBP, central SBP; MAP, mean arterial pressure. *P* value derived from Anova test.

^*^P < 0.05 compared with White UK.

^**^P < 0.01 compared with White UK.

**TABLE 2:** Augmentation index at 21–23 years: regression models for the influence of blood pressure, height, heart rate, sex and social exposures from early adolescence. The Determinants of Adolescent, now young Adults, Social well-being and Health study

	Core model^a^			+ Socioeconomical variables^b^			+Childhood variables^c^		
Variables	Coeff.	95% CI	*P* value	Coeff.	95% CI	*P* value	Coeff.	95% CI	*P* value
Sex (Male – Ref)									
Female	3.53	(1.67, −5.39)	<0.001	3.73	(1.80–5.70)	<0.001	5.37	(2.80–8.00)	<0.001
Pulse	−0.28	(−0.35, −0.22)	<0.001	−0.31	(−0.40, –0.20)	<0.001	−0.31	(−0.40, –0.20)	<0.001
Central SBP	0.43	(0.38, 0.49)	<0.001	0.43	(0.40, 0.50)	<0.001	0.41	(0.30, 0.30)	<0.001
Height	−0.36	(−0.45, −0.26)	<0.001	−0.39	(−0.50, –0.30)	<0.001	−0.23	(−0.40, –0.90)	0.002
Ethnicity (White UK – Ref)									
Black Caribbean	1.32	(−0.99, 3.63)	0.260	0.91	(−1.50, 3.30)	0.460	0.87	(−1.70, 3.50)	0.507
Black African	1.73	(−0.39, −3.85)	0.110	1.36	(−0.90, 3.60)	0.240	2.65	(0.03–5.30)	0.047
Indian	3.35	(1.02, 5.67)	0.005	3.00	(0.60, 5.40)	0.015	3.97	(1.40, 6.60)	0.003
Pakistani/Bangladeshi	4.20	(1.89, 6.52)	<0.001	4.60	(2.10, 7.10)	<0.001	4.34	(1.60, 7.10)	0.002
Others	1.17	(−1.03, 3.37)	0.590	0.96	(−1.70, 3.30)	0.420	1.29	(−1.30, 3.90)	0.324
Reported racism (No – Ref)									
Yes				0.89	(−0.80, 2.60)	0.315			
Own employment (Yes – Ref)									
No				−0.50	(−1.90, 0.90)	0.470			
Education (No – Ref)									
Yes				–0.92	(–2.3, 0.50)	0.194			
MAP (11–13 years)							−0.11	(−0.20, –0.01)	0.055
Height (11–13 years)							−0.09	(−0.20, −0.30)	0.130
Reported racism (No – Ref)									
Yes							0.69	(−0.90, 2.40)	0.430
Family affluence scale 5(≥3 – Ref)									
1–2							0.25	(−1.40, 1.90)	0.760
1–3							2.82	(−0.90, 7.50)	0.340
Parental employment (Yes – Ref)									
No							−0.47	(−3.01, 1.30)	0.429

CI, confidence interval; MAP, mean arterial pressure.

^a^Core model: AIx adjusted for age, heart rate, height, central BP at 21–23 years, sex, and ethnicity.

^b^Core model + socioeconomic circumstances at 21–23 years: reported racism, own employment, and education.

^c^Core model + childhood variables (11–13 years): MAP, height, reported racism at 11–13 years, family affluent scale at 11–13 years, per annum.

## Reviewers’ Summary Evaluations

### Referee 1

Strengths: 1. Population-based sample participating in the Determinants of Adolescent Social well being and Health (DASH); 2. The study assesses central SBP and augmentation index as potential precursors of greater risk in minority populations vs. the white majority; 3. The authors identify clear differences in augmentation index between South Asian populations and White UK youth.

Weaknesses: 1. Relatively limited number of youth in each group studied (∼100); 2. Absence of target organ data, e.g. LVH, carotid intimal–media thickness. Clinical events would not be expected in this young population.
